# Geschmacksneutrale Andickungsmittel? – Ein kompetitiver Vergleich

**DOI:** 10.1007/s00106-022-01161-1

**Published:** 2022-04-27

**Authors:** Steffen Schulz, Veronika Scholz, Bernhard Lehnert

**Affiliations:** 1 FB Angewandte Gesundheitswissenschaften, Europäische Fachhochschule, Rostock, Deutschland; 2 Klinik und Poliklinik für Neurologie, Universitätsmedizin Greifswald, Greifswald, Deutschland; 3 Klinik und Poliklinik für Hals‑, Nasen‑, Ohrenheilkunde, Kopf- und Halschirurgie, Abteilung Phoniatrie und Pädaudiologie, Universitätsmedizin Greifswald, Ferdinand-Sauerbruch-Str., 17475 Greifswald, Deutschland

**Keywords:** Dysphagie, Diät, Kost und Ernährung, Trinkverhalten, Geschmack, Geschmackswahrnehmung, Dysphagia, Diet, food, and nutrition, Drinking behavior, Taste, Taste perception

## Abstract

**Hintergrund:**

Das Andicken von Flüssigkeiten gehört zu den Standardverfahren der Dysphagietherapie. Diese adaptive Methode soll u. a. einem posterioren Leaking entgegenwirken und die Anforderung an verlangsamte Schutzreflexe durch eine reduzierte Fließgeschwindigkeit des Bolus senken. Bisherige Erhebungen zeigen jedoch aufgrund der Geschmacksperzeption eine ablehnende Haltung von Patienten gegenüber angedickten Flüssigkeiten. Diese Studie untersucht, ob zwischen verschiedenen Andickungsmitteln Geschmacksunterschiede bestehen.

**Methoden:**

An der Studie haben 37 gesunde Probanden Teil genommen und 8 auf dem deutschen Markt erhältliche Andickungsmittel untereinander verglichen. Zur Testung wurden jeweils 2 mit Wasser angerührte Andickungsmittel einander gegenübergestellt. Die Probanden sollten dann entscheiden, welches sie geschmacklich präferierten. Bis zu 7 dieser Paarvergleiche wurden von jedem Probanden vorgenommen. Insgesamt wurden 224 Paarvergleiche durchgeführt. Aus diesen wurde mittels eines probabilistischen Modells eine relative Geschmacksgüte bestimmt und eine Signifikanztestung der Unterschiede durchgeführt.

**Ergebnisse und Schlussfolgerung:**

Zwischen den verschiedenen Andickungsmitteln zeigten sich signifikante Geschmacksunterschiede. Es kann vermutet werden, dass sich die Geschmacksunterschiede auf die Inhaltsstoffe der jeweiligen Andickungsmittel zurückführen lassen. Im therapeutischen Setting sollte für eine höhere Akzeptanz von Kostanpassungen nach Möglichkeit die Ausprobe unterschiedlicher Andickungsmittel erfolgen. Unklar bleibt, ob die hier gezeigten Geschmacksunterschiede sich auch zeigen, wenn anstelle von Wasser andere Flüssigkeiten wie Kaffee, Tee oder Säfte angedickt werden.

## Abstract (en)

**Background:**

Thickening of drinks is a standard procedure in dysphagia therapy. Among other things, this adaptive procedure aims to prevent posterior leakage and reduce the demands placed on retarded swallowing reflexes by decelerating boluses. Studies show that taste perception can induce a negative attitude towards thickened fluids in patients. This study investigates whether different thickeners differ in taste.

**Methods:**

The taste of eight arbitrarily chosen thickeners available on the German market were compared by 37 healthy adults. In the test setting, two thickeners combined with water competed against each other. Participants decided which one they preferred. Up to seven pairwise comparisons were performed by each participant. Overall, 224 comparisons were carried out. Based on these results, a relative taste grade was calculated using a probabilistic model and significance tests for differences were performed.

**Results and conclusion:**

There are significant differences in taste between the different products, presumably depending on their respective basic ingredients. To respect individual patient’s preferences, different thickeners should be tried out in dysphagia therapy. It remains unclear whether thickeners’ taste differences remain relevant once other liquids such as coffee, tea, or juice are thickened.

Eine Dysphagie kann durch verschiedene Probleme im oralen, pharyngealen, laryngealen oder ösophagealen Bereich verursacht sein. Unbehandelt kann sie zu Mangelernährung, Exsikkose und Aspirationspneumonien führen [1]. Weitere Folgen können Partizipationseinschränkungen und ein Rückgang der Lebensqualität sein [9]. Nicht zuletzt können Gesundheitsschäden durch eine behinderte Tabletteneinnahme auftreten [4].

Neben dem Einsatz restituierender und kompensatorischer Therapieansätze bestehen im therapeutischen Management auch Möglichkeiten der Kostadaption. Die Veränderung rheologischer Eigenschaften durch die Verwendung von Andickungsmitteln impliziert den Gedanken, die Boluspassage sicherer zu gestalten [26]. Beispielsweise kann damit einem vorzeitigen Abgleiten von Bolusanteilen in den Pharynx entgegengewirkt werden [19]. Die Reduktion des Aspirationsrisikos durch das Andicken von Getränken heben Steele et al. [25] in einem Review sowie Newman et al. [21] in einem White Paper der European Society for Swallowing Disorders (ESSD) hervor.

Jedoch ist der mittel- bis langfristige Benefit angedickter Flüssigkeiten in der Versorgung dysphagischer Patienten umstritten. Mehrere Untersuchungen belegen, dass Personen, die angedickte Getränke zu sich nehmen, eine geringere Flüssigkeitsaufnahme aufweisen als vergleichbare Gruppen ohne Diätmodifikation [4, 6, 20, 28].

Bock et al. [2] haben in einer retrospektiven Untersuchung der Akten von 564 Aspirationspatienten keinen Einfluss von therapeutischer Kostadaptation einschließlich Andicken auf die Häufigkeit pulmonaler Komplikationen (genauer: den Zeitraum bis zum Eintreten des ersten pulmonalen Ereignisses) oder die Mortalität gefunden. Sie diskutieren als Schwäche ihrer Arbeit, dass die tatsächliche Einhaltung der Ernährungsempfehlungen nicht dokumentiert wurde.

Wenn die Ziele der Ernährungsempfehlungen (einschließlich Andickungsmitteln) nicht erreicht werden, stellt sich die Frage, ob diese Empfehlungen von vielen Patienten gar nicht umgesetzt werden. Die Veränderung eines Getränks durch Andickungsmittel kann einen Einfluss auf das Geschmacksempfinden haben. Eine daraus resultierende Ablehnung konnte bereits in mehreren Untersuchungen nachgewiesen werden [5, 18].

Ein die Patientencompliance beeinflussender Faktor liegt vermutlich in der individuellen Auswahl eines Andickungsmittels und den Anteilen seiner Bestandteile wie z. B. modifizierte Maisstärke, Maltodextrin oder Xanthan. Dies liegt nahe, da in verschiedenen Studien durch sowohl gesunde Personen als auch Patienten signifikante Unterschiede in der Geschmacksbeurteilung von Andickungsmitteln detektiert wurden [14–17].

Die Akzeptanz der Betroffenen ist eine essenzielle Voraussetzung für den Erfolg dieser Therapiemaßnahme. Incompliance kann eine Unterversorgung des Wasserhaushalts nach sich ziehen [10]. Denkbar ist, dass Betroffene Flüssigkeiten ohne Texturveränderungen zu sich nehmen und damit Aspirationen und Pneumonien provozieren.

Das Ziel dieser Studie ist es daher, eine Auswahl an auf dem deutschen Markt erhältlichen Andickungsmitteln hinsichtlich ihrer geschmacklichen Einschätzung durch gesunde Probanden zu untersuchen.

## Methodik

### Stichprobe

Siebenunddreißig in der Selbstauskunft gesunde Studierende (2.–6. Semester) der Fachrichtung Logopädie an der Europäischen Fachhochschule in Rostock nahmen an der Studie Teil (1 Mann und 36 Frauen). Das Durchschnittsalter betrug 21,5 (±1,8, Range 18–28) Jahre.

Ausschlusskriterien waren akute oder chronische Rhinitis sowie bekannte Störungen des Schmeckens und Riechens. Alle Daten wurden anonym erhoben.

### Material

Acht unterschiedliche Andickungsmittel (Tab. 1) wurden in stillem Mineralwasser der Marke „Gut & Günstig“ gelöst. Dies erfolgte manuell mithilfe von Schüttelbechern der Firma „Nutricia“, die speziell für das Andicken von Flüssigkeiten vorgesehen sind (Volumen max. 250 ml). Es wurden einzelne Schmeckproben zu je 5 ml in Einwegspritzen abgefüllt und den Probanden zur eigenständigen Verkostung übergeben.

**Table 1:** 

Probennummer	Präparat	Ingredienzien
A1	Nutilis Clear (Nutricia Milupa GmbH, Erlangen, Deutschland)	Maltodextrin, Xanthan, Guarkernmehl
A2	Fresubin Clear Thickener (Fresenius Kabi, Bad Homburg, Deutschland)	Modifizierte Stärke, Xanthan, Maltodextrin, modifizierte Zellulose
A3	Nutilis Powder	Maltodextrin, Tarakernmehl, Xanthan, Guarkernmehl
A4	ThickenUp Clear (Nestlé Health Science S.A., Vevey, Schweiz)	Maltodextrin, Xanthan, Kaliumchlorid
A5	JONOVA Andickungspulver klar (JONOVA, Heilbronn, Deutschland)	Maltodextrin, Xanthan, Palmöl
A6	ThickenUp	Modifizierte Maisstärke
A7	JONOVA Andickungspulver	Kartoffelstärke
A8	Thick & Easy (Fresenius Kabi, Bad Homburg, Deutschland)	Modifizierte Maisstärke, Maltodextrin

### Versuchsaufbau und Durchführung

Die Geschmacksvergleiche der Andickungsmittel erfolgten in mehreren jeweils paarweisen Gegenüberstellungen. Die Probanden sollten sich wiederholt entscheiden, welche von je zwei Proben ihnen besser schmeckten.

Acht Andickungsmittel ermöglichen 56 verschiedene Paarungen, wenn man die Reihenfolge, wie hier geschehen, vorgibt. Mithilfe des Computerprogramms R [27] wurde vor Versuchsbeginn für jeden Probanden ein individuell randomisierter Versuchsplan mit je bis zu sieben Paarvergleichen erstellt. Bei der Randomisierung wurde beachtet, dass es innerhalb eines individuellen Versuchsplans nicht zu einer Mehrfachbeurteilung der gleichen Kombination kam.

Die Datenerhebung erfolgte aus logistischen Gründen zu drei Testzeitpunkten. Zu jedem Zeitpunkt unterzog sich eine andere Gruppe von Probanden dem Versuch. Während der Testung waren die Teilnehmer hinsichtlich der Präparate verblindet.

Da unklar war, wie viele Andickungsmittelproben den Probanden zumutbar waren, wurde ihnen freigestellt, die Teilnahme jederzeit zu beenden. Um übermäßige Einflussnahme durch einzelne Probanden zu vermeiden, wurde die Zahl der Geschmacksurteile pro Proband auf sieben begrenzt.

Eine Stunde vor jedem der drei Messzeitpunkte wurden entsprechend der Herstellerangaben jeweils 200 ml stilles Mineralwasser auf der vorgegebenen Andick-Stufe 1 (sirup- bzw. nektarartig) angedickt. Die Schüttelbecher wurden dafür 50-mal mit alternierenden Auf- und Abwärtsbewegungen manuell geschüttelt. Anschließend wurden die Proben in Einwegspritzen aufgezogen und in Paaren für die individuellen Versuchspläne bereitgestellt.

Für die Testung der Präparate spritzten sich die Teilnehmer zunächst die erste Probe des Paares unter die Zunge und schluckten sie herunter. Im Anschluss wurde ebenso mit der zweiten Probe verfahren. Die Probanden haben dann auf dem Versuchsplan vermerkt, welche Probe ihnen besser schmeckte. Zwischen den Paarvergleichen war es den Probanden freigestellt, mit bereitgestelltem Wasser den Mundraum zu neutralisieren. Die Versuchsdurchläufe erstreckten sich jeweils über einen Zeitraum von ca. 20 min.

## Datenauswertung und Statistik

### Statistische Auswertung von Paarvergleichen

Zur Auswertung der Paarvergleiche wurde ein probabilistisches Modell nach Bradley und Terry [3] erstellt. Dabei wird angenommen, dass jedes Andickungsmittel eine als Zahl ausdrückbare *wahre Geschmacksgüte* π hat. Im Vergleich zweier Andickungsmittel hängt die Chance, als „besser schmeckend“ eingestuft zu werden, vom Verhältnis der beiden π‑Werte ab. Im Rahmen einer logistischen Regression wird für jedes Andickungsmittel A_i_ ein Koeffizient β_i_ geschätzt. Aus der Differenz zweier Koeffizienten β_i_ und β_j_ lässt sich die Wahrscheinlichkeit p_ij_ bestimmen, dass A_i_ gegenüber A_j_ als wohlschmeckender empfunden wird: logit(p_ij_) = β_i_ − β_j_. Die β‑Koeffizienten bilden eine Rangfolge der Andickungsmittel nach Geschmack. Je größer β_i_, desto besser hat das zugehörige Andickungsmittel A_i_ den Teilnehmern geschmeckt. Zusätzlich wurden (Quasi‑)Standardfehler für die verschiedenen β‑Koeffizienten gemäß [11] bestimmt.

Für den statistischen Vergleich wurde Andickungsmittel A_1_ mit β_1_ = 0 definiert, da die Geschmacksgüte keinen natürlichen Nullpunkt besitzt. In weiteren Regressionsrechnungen wurde jedes Präparat einmal als Referenzkategorie festgelegt, damit alle Andickungsmittel vergleichend einander gegenübergestellt werden können. Auf diese Weise kann für jeden Paarvergleich eine Signifikanzprüfung durchgeführt werden.

Zur Absicherung gegen einen Reihenfolgeeffekt in Abhängigkeit davon, ob Proben von den Teilnehmern als Erstes oder als Zweites probiert wurden, wurde ein Log-Likelihood-Test durchgeführt. Das Alphaniveau wurde auf 0,05 festgelegt. Alle Berechnungen wurden mit R [12, 27] durchgeführt.

## Ergebnisse

### Deskriptive Statistik

**Figure 1:**
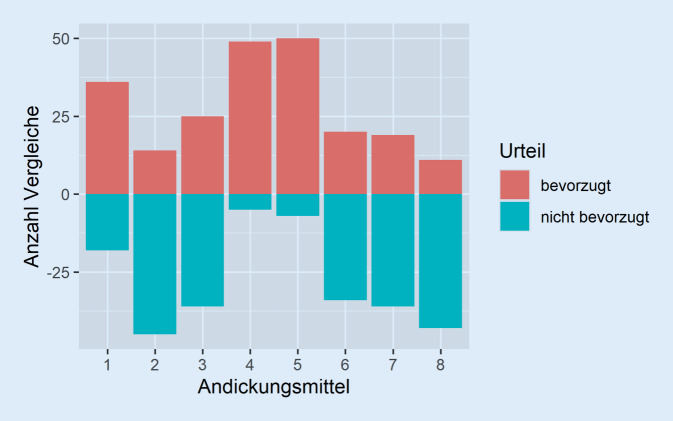

Insgesamt nahmen die 37 Teilnehmer 224 Paarvergleiche vor. Die Tab. 2 zeigt die Häufigkeitsverteilung der durchgeführten Vergleiche. Die Häufigkeit der jeweils präferierten Andickungsmittel innerhalb der Paarvergleiche ist in Tab. 3 gegeben. Die Abb. 1 visualisiert, wie häufig ein Andickungsmittel insgesamt bevorzugt und nicht bevorzugt wurde.

**Table 2:** 

		Als Zweites getestet							
		A1	A2	A3	A4	A5	A6	A7	A8
Als Erstes getestet	A1	–	5	5	3	4	3	4	3
	A2	3	–	4	4	5	4	4	4
	A3	4	5	–	5	4	4	4	5
	A4	4	5	5	–	2	5	3	3
	A5	5	5	5	3	–	4	4	3
	A6	3	4	4	4	4	–	5	4
	A7	4	4	3	5	4	2	–	4
	A8	4	3	4	3	5	4	5	–

**Table 3:** 

		Nicht bevorzugt							
		A1	A2	A3	A4	A5	A6	A7	A8
Bevorzugt	A1	–	7	6	2	2	6	7	6
	A2	1	–	3	0	0	3	3	4
	A3	3	6	–	1	0	3	5	7
	A4	5	9	9	–	4	9	7	6
	A5	7	10	9	1	–	7	8	8
	A6	0	5	5	0	1	–	4	5
	A7	1	5	2	1	0	3	–	7
	A8	1	3	2	0	0	3	2	–

### Probabilistisches Modell – Gesamtsignifikanz

Die Tab. 4 zeigt die Ergebnisse der Bradley-Terry-Regression mit Andickungsmittel 1 als (willkürlich gewählter) Referenzkategorie und mit dummykodierter Untersuchung auf einen Reihenfolgeeffekt. Das Modell ist im Log-Likelihood-Test mit *p* < 0,01 signifikant. Die visuell gezeigten Effekte der Auswahl des Andickungsmittels sind also überzufällig.

**Table 4:** 

	β	Standardfehler	*p*-Wert	Quasi-Standardfehler
A1	0	–	–	0,37
A2	−2,20	0,50	< 0,01	0,32
A3	−1,35	0,47	< 0,01	0,29
A4	1,49	0,59	0,01	0,52
A5	1,19	0,53	0,03	0,46
A6	−1,61	0,49	< 0,01	0,31
A7	−1,73	0,49	< 0,01	0,31
A8	−2,52	0,52	< 0,01	0,34
Erster	0,13	0,18	0,49	–

### Probabilistisches Modell – Paarvergleiche

**Figure 2:**
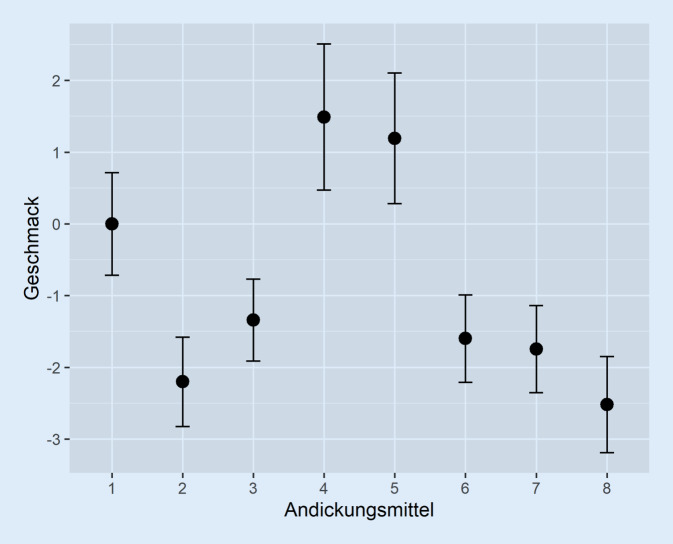

Die Tab. 4 gibt einen Überblick der *p*-Werte und Quasi-Standardfehler aller Präparate mit Bezug zur Referenzkategorie A1. In Tab. 5 sind die Ergebnisse der Regressionsberechnungen dargestellt, bei denen jeweils ein Andickungsmittel als Referenzkategorie gilt. Die Geschmackskoeffizienten β der Andickungsmittel und deren 1,96-fache Quasi-Standardfehler lassen sich in Referenz zu A1 (β = 0) aus Abb. 2 entnehmen.

**Table 5:** 

	Andickungsmittel							
	A1	A2	A3	A4	A5	A6	A7	A8
A1	–	< 0,01	< 0,01	0,01	0,03	0,01	< 0,01	< 0,01
A2	< 0,01	–	< 0,05	< 0,01	< 0,01	0,17	0,28	0,48
A3	< 0,01	0,46	–	< 0,01	< 0,01	0,54	0,37	< 0,01
A4	0,01	< 0,01	< 0,01	–	0,61	< 0,01	< 0,01	< 0,01
A5	0,03	< 0,01	< 0,01	0,61	–	< 0,01	< 0,01	< 0,01
A6	0,001	0,17	0,53	< 0,01	< 0,01	–	0,78	0,04
A7	< 0,01	0,28	0,37	< 0,01	< 0,01	0,78	–	0,08
A8	< 0,01	0,48	< 0,01	< 0,01	< 0,01	0,04	0,08	–

### Probabilistisches Modell – Reihenfolgeeffekt

Ein signifikanter Reihenfolgeeffekt liegt nicht vor (*p* = 0,48).

## Diskussion

Da das probabilistische Modell als Ganzes signifikant ist, ist ein systematischer Einfluss bedingt durch die Geschmacksgüte der Andickungsmittel gezeigt. Ein Reihenfolgeeffekt bei der Verkostung zeigt sich nicht.

Im Vergleich der einzelnen Präparate gegeneinander bestehen mitunter sehr deutliche Unterschiede in der Geschmacksgüte. Dies ist im Einklang mit anderen Untersuchungen zur Geschmacksbeurteilung angedickter Flüssigkeiten [14–17]. Besonders häufig werden, wie Abb. 1 zu entnehmen ist, die Präparate A4 und A5 gegenüber anderen präferiert. Die Abb. 2 sowie Tab. 5 lassen erkennen, dass sich diese beiden sowie A1 jeweils signifikant von allen anderen abgrenzen. Zwar enthalten alle drei Produkte Ballaststoffe wie Xanthan, jedoch ist dieser Inhaltsstoff ebenfalls in A2 und A3 enthalten.

Die kausalen Einflussfaktoren, die zur unterschiedlichen Geschmacksbeurteilung führen, kann diese Studie nicht aufklären. Es wurde vor allem gezeigt, dass keine geschmackliche Neutralität über alle Produkte hinweg besteht.

Die von Kritikern der diätetischen Modifikation mit Andickungsmitteln angeführte geringere Flüssigkeitsaufnahme [4, 6, 20, 29] könnte daher in einer verminderten (geschmacklichen) Akzeptanz der verwendeten Präparate begründet sein. Studien, die beispielweise die Flüssigkeitsaufnahme als Endpunkt haben, sollten ihre Ergebnisse hinsichtlich der verwendeten Andickungsmittel und deren Bestandteilen kontrollieren.

Die in dieser Arbeit aufgedeckten Unterschiede in der geschmacklichen Bewertung zwischen den Produkten unterstützten die Empfehlung der Leitlinie neurogener Dysphagien [8], verschiedene Andickungsmittel mit den Patienten auszuprobieren. Zusätzlich lassen die Ergebnisse in Tab. 3 vermuten, dass ebenso Unterschiede zwischen individuellen Präferenzen bestehen. So wird zum Beispiel sechsmal A1 gegenüber A3 bevorzugt, jedoch existiert dreimal auch der umgekehrte Fall. (In unserem statistischen Modell werden auch die Vergleiche beider Andickungsmittel mit den jeweils anderen herangezogen, sodass dennoch eine statistisch signifikante Überlegenheit von A1 gezeigt werden konnte).

Eine verbesserte Compliance von Patienten gegenüber der Verwendung von Andickungsmitteln kann möglicherweise negative Folgen [4, 6, 10, 20, 24, 29] limitieren und den implizierten Benefit [21, 25, 26] stärker herausstellen.

### Limitationen

Die alleinige Testung mit Wasser in Kombination mit den Andickungsmitteln erlaubt den Schluss, dass die Andickungsmittel unterschiedlich und damit keinesfalls alle geschmacksneutral sind. Der Eigengeschmack anderer Flüssigkeiten wie Tee, Kaffee, Fruchtsäfte oder Brühe mag in der klinischen Realität die für Wasser gefundenen Unterschiede nihilieren oder gar umkehren. Das wurde in der vorliegenden Studie nicht untersucht.

Auch ist die in dieser Arbeit untersuchte junge, gesunde und fast ausschließlich weibliche Stichprobe sehr wahrscheinlich nicht repräsentativ für überwiegend ältere Patienten mit Dysphagie.

Die Testung der Probanden in drei Gruppen war dem logistischen Vorteil eines geringeren Vorbereitungsaufwands beim Präparieren der Proben geschuldet. Jeder Proband folgte einem individuellen Versuchsplan und war aufgefordert, die Beurteilung nach außen hin neutral zu gestalten. Dennoch kann ein gegenseitiges Beeinflussen der Probanden untereinander nicht ausgeschlossen werden. Aufgrund der Paralleltestung der Probanden einer Gruppe wurden die Getränke bereits eine Stunde vor der Testung angedickt, da Zeit zum Aufziehen der Flüssigkeiten auf die Einwegspritzen benötigt wurde. Es ist davon auszugehen, dass die Konsistenz von mit Wasser versetzten Andickungsmitteln sich im Zeitverlauf verändert [7, 13, 22]. Besonders Produkte mit Maltodextrin als Basis scheinen hierfür empfänglich zu sein [7]. Hierin kann ein Störfaktor liegen, da zu stark angedickte Getränke auch stärker abgelehnt werden [23]. Dies wurde in der vorliegenden Studie nur durch die Randomisierung der Versuchsreihenfolge angegangen.

## Ausblick

Die Untersuchung zeigt Unterschiede in der geschmacklichen Beurteilung von Andickungsmitteln im deutschen Markt. Zur Untermauerung der Ergebnisse und der Ausweitung des klinischen Nutzens können sich zukünftige wissenschaftliche Arbeiten an der vorliegenden Auswertungsmethodik orientieren. Beispielsweise könnte der Einschluss tatsächlich betroffener Probanden, einer alltagsgerechten Beachtung der sich über die Zeit verändernden Fließeigenschaften von Angedicktem und eine noch umfangreichere Auswahl an Präparaten in zukünftigen Studien zu konkreteren Empfehlungen für die Patienten führen.

Darüber hinaus sollten zukünftige Studien auch der generellen Akzeptanz gegenüber Andickungsmitteln nachgehen und hierbei Umfang und Dauer der diätetischen Modifikation berücksichtigen.

## Fazit für die Praxis

- Andickungsmittel sind nicht generell geschmacksneutral.
- Auch wenn mal das eine und mal das andere Andickungsmittel als besser schmeckend gekennzeichnet wurde, ergeben sich in der statistischen Auswertung solche, die meistens besser, und solche, die meistens schlechter schmeckten.
- Fällt die Entscheidung für das Andicken von Flüssigkeiten, sollte nach Möglichkeit mit jedem Patienten individuell eine Auswahl an Präparaten erprobt werden, um individuelle Präferenzen erkennen zu können.
